# Supplementary material for: Overexpression of Egr1 Transcription Regulator Contributes to Schwann Cell Differentiation Defects in Neural Crest-Specific Adar1 Knockout Mice
Source: Cells. 2024 Nov 23;13(23):1952. doi: 10.3390/cells13231952 (PMC11639873; doi:10.3390/cells13231952)
Supplement: Supplementary file 1 [file cells-13-01952-s001.zip › cells-3281069-supplementary.pdf]

**Supplementary Materials**

## Supplemental Tables

**Table S1:** List of primers sequences forward and reverse from 5'-3' used for RT-qPCR

| RT-qPCR primers | | | | |
|---|---|---|---|---|
| **Genes** | | | | |
| *Rxrg* | Forward | TGAACTACCCATCCACCAGC | Reverse | GGCACTTCTGATAGCGACA |
| *Fosl2* | Forward | AGATGAGCAGCTGTCTCCTG | Reverse | GCTTGATGACAGAGCGCT |
| *Pml* | Forward | TTTCCGTGCAGAAGGTACCT | Reverse | GAACCAGTTTCCGTGCAGAA |
| *Ddit3* | Forward | CACCTGAAAGCAGAACCTGG | Reverse | GGACGCAGGGTCAAGAGTAG |
| *Tfap2b* | Forward | ACTTAGCTCGGGATTTCGGG | Reverse | AGACTGAAGTGCGTGAGACA |
| *Fosl1* | Forward | CTCATTTCCTGGGGCCCA | Reverse | ATTTTGCAGATGGGTCGGTG |
| *Cx32* | Forward | GTACCCCACTTTGGGAGTCA | Reverse | TCCGATTTATCTGCCTGCTT |
| *Prx* | Forward | TGAGTGCCCGTGTGTTCTTT | Reverse | GCGCTTCAAGCAGAAGGAGA |
| *Chka* | Forward | GGCCAAGATCTCATCCATTGAA | Reverse | TGGTCAAAGTAGGCCTCGAATC |
| *Scd1* | Forward | CCTCTGGAGCCACAGAACTT | Reverse | GCCATGGTGTTGGCAATGAT |
| *Cyp51* | Forward | ACGCTGCCTGGCTATTGC | Reverse | TTGATCTCTCGATGGGCTCTATC |
| *Hmgcr* | Forward | AACTGGTGCAGAAATCTCTAGC | Reverse | GGTTGAATAGCTCAGAACTAGCC |
| *Dhcr7* | Forward | CAAGACACCACCTGTGACAGCT | Reverse | CTGCTGGAGTAATGGCACCTTC |
| *Mag* | Forward | GTTTGCCCCCATAATCCTTCTG | Reverse | TCCCTCTCCGTCTCATTCACAGTC |
| *S100b* | Forward | GCTGAGCAAGAAAGAACTGAA | Reverse | AGCCACCAGCACAACATAC |
| *Pou3f1* | Forward | TTCAAGCAACGACGCATCAA | Reverse | TGCGAGAACACGTTACCGTAGA |

**Table S2**: List of 180 transcriptional regulators found in common in RNA-seq 1 and in RNA-seq 2 data sets (with FC >2)

| | | 1st RNA-seq | | 2nd RNA-seq | |
|---|---|---|---|---|---|
| Gene symbol | Full name | Fold change | P-value | Fold change | P-value |
| Chp2 | calcineurin-like EF hand protein 2 | 0,13 | 2,03E-34 | 0,07 | 6,84718E-37 |
| Abca2 | ATP-binding cassette, sub-family A (ABC1), member 2 | 0,14 | 2,20E-39 | 0,22 | 1,37891E-09 |
| Wnt6 | wingless-type MMTV integration site family, member 6 | 0,16 | 3,81E-35 | 0,40 | 0,000136894 |
| Lmo1 | LIM domain only 1 | 0,17 | 9,28E-05 | 0,19 | 2,63034E-09 |
| Ldlr | low density lipoprotein receptor | 0,2 | 4,14E-54 | 0,33 | 1,317E-09 |
| Cdh1 | cadherin 1 | 0,2 | 1,31E-02 | 0,18 | 8,95745E-06 |
| Mitf | microphthalmia-associated transcription factor | 0,2 | 6,09E-14 | 0,28 | 0,000494504 |
| Ednrb | endothelin receptor type B | 0,22 | 3,76E-53 | 0,32 | 7,48202E-07 |
| Plp1 | proteolipid protein (myelin) 1 | 0,22 | 1,10E-31 | 0,28 | 1,39242E-07 |
| Rxrg | retinoid X receptor gamma | 0,23 | 8,93E-23 | 0,18 | 1,1578E-15 |
| Pou3f1 | POU domain, class 3, transcription factor 1 | 0,23 | 1,62E-24 | 0,30 | 1,62105E-08 |
| Rps6ka2 | ribosomal protein S6 kinase, polypeptide 2 | 0,24 | 4,47E-21 | 0,29 | 5,147E-07 |
| Mapre3 | microtubule-associated protein, RP/EB family, member 3 | 0,24 | 2,05E-24 | 0,32 | 3,90711E-07 |
| Bmp7 | bone morphogenetic protein 7 | 0,26 | 3,59E-13 | 0,30 | 3,48314E-10 |
| Cux1 | cut-like homeobox 1 | 0,28 | 6,05E-25 | 0,41 | 0,00190925 |
| Pf4 | platelet factor 4 | 0,29 | 1,45E-21 | 0,18 | 9,41636E-05 |
| Satb1 | special AT-rich sequence binding protein 1 | 0,3 | 4,22E-12 | 0,28 | 2,17096E-11 |
| Cntn1 | contactin 1 | 0,3 | 1,01E-12 | 0,20 | 2,04809E-10 |
| S100a1 | S100 calcium binding protein A1 | 0,31 | 4,99E-22 | 0,18 | 2,06644E-10 |
| Cdkn1c | cyclin-dependent kinase inhibitor 1C (P57) | 0,32 | 1,02E-19 | 0,25 | 6,32991E-08 |
| Mxd4 | Max dimerization protein 4 | 0,33 | 1,20E-40 | 0,37 | 8,26742E-13 |
| Arnt2 | aryl hydrocarbon receptor nuclear translocator 2 | 0,33 | 1,34E-10 | 0,36 | 0,000810952 |
| Crebl2 | cAMP responsive element binding protein-like 2 | 0,33 | 5,22E-11 | 0,42 | 1,06238E-06 |
| Pcx | pyruvate carboxylase | 0,34 | 3,26E-02 | 0,32 | 7,35847E-10 |
| Sirt2 | sirtuin 2 | 0,34 | 1,98E-19 | 0,38 | 1,45367E-10 |
| Itga6 | integrin alpha 6 | 0,35 | 4,78E-22 | 0,49 | 0,000101818 |
| Foxo4 | forkhead box O4 | 0,35 | 1,04E-15 | 0,47 | 1,40326E-06 |
| Tead4 | TEA domain family member 4 | 0,35 | 1,21E-04 | 0,42 | 0,00046445 |
| Hdac11 | histone deacetylase 11 | 0,35 | 2,70E-11 | 0,37 | 1,14622E-07 |
| Mt3 | metallothionein 3 | 0,36 | 2,57E-05 | 0,14 | 8,68786E-09 |
| Nacc2 | nucleus accumbens associated 2, BEN and BTB (POZ) domain containing | 0,37 | 7,23E-15 | 0,43 | 0,000108766 |
| Cryab | crystallin, alpha B | 0,38 | 4,71E-14 | 0,32 | 9,70727E-05 |
| Pink1 | PTEN induced putative kinase 1 | 0,38 | 2,03E-25 | 0,38 | 9,42704E-07 |
| Mlip | muscular LMNA-interacting protein | 0,38 | 2,09E-17 | 0,24 | 7,54258E-07 |
| Kras | Kirsten rat sarcoma viral oncogene homolog | 0,38 | 2,96E-24 | 0,37 | 8,611E-10 |
| Ndfip2 | Nedd4 family interacting protein 2 | 0,43 | 4,52E-13 | 0,39 | 1,2006E-08 |
| Ndp | Norrie disease (pseudoglioma) (human) | 0,43 | 6,41E-04 | 0,19 | 2,72185E-09 |
| Med12l | mediator complex subunit 12-like | 0,43 | 5,17E-12 | 0,44 | 0,0111337 |
| Myo6 | myosin VI | 0,44 | 9,97E-11 | 0,33 | 2,37983E-09 |
| Slc40a1 | solute carrier family 40 (iron-regulated transporter), member 1 | 0,44 | 9,82E-05 | 0,33 | 3,52664E-05 |
| Taf13 | TATA-box binding protein associated factor 13 | 0,44 | 6,13E-17 | 0,42 | 0,000153922 |

| | | | | | |
|---|---|---|---|---|---|
| Ccdc85b | coiled-coil domain containing 85B | 0,45 | 4,15E-17 | 0,40 | 4,56285E-05 |
| Wwp1 | WW domain containing E3 ubiquitin protein ligase 1 | 0,45 | 5,81E-13 | 0,32 | 1,66815E-07 |
| Hes6 | hairy and enhancer of split 6 | 0,46 | 1,30E-02 | 0,40 | 0,00017627 |
| Acvr2b | activin receptor IIB | 0,47 | 3,63E-09 | 0,49 | 2,58661E-05 |
| Nfkbiz | nuclear factor of kappa light polypeptide gene enhancer in B cells inhibitor, zeta | 2,02 | 1,65E-05 | 2,34 | 0,000108672 |
| Aif1 | allograft inflammatory factor 1 | 2,04 | 1,16E-05 | 2,81 | 0,007901834 |
| Zfp217 | zinc finger protein 217 | 2,06 | 1,71E-07 | 2,13 | 1,29565E-09 |
| Lbh | limb-bud and heart | 2,10 | 2,56E-14 | 2,40 | 2,13819E-08 |
| Pprc1 | peroxisome proliferative activated receptor, gamma, coactivator-related 1 | 2,12 | 1,48E-14 | 2,38 | 2,20134E-09 |
| Sbno2 | strawberry notch homolog 2 (Drosophila) | 2,21 | 2,02E-16 | 2,30 | 4,01286E-06 |
| Nr1d1 | nuclear receptor subfamily 1, group D, member 1 | 2,27 | 2,38E-08 | 2,29 | 7,8723E-08 |
| Havcr2 | hepatitis A virus cellular receptor 2 | 2,28 | 9,60E-04 | 3,12 | 0,001866767 |
| Rara | retinoic acid receptor, alpha | 2,28 | 1,42E-16 | 2,37 | 2,02428E-16 |
| Sap30 | sin3 associated polypeptide | 2,28 | 5,51E-11 | 2,02 | 0,01902268 |
| Mafk | v-maf musculoaponeurotic fibrosarcoma oncogene family, protein K (avian) | 2,28 | 1,55E-17 | 2,26 | 6,7839E-10 |
| Elf4 | E74-like factor 4 (ets domain transcription factor) | 2,30 | 4,26E-07 | 2,48 | 7,22031E-07 |
| Akna | AT-hook transcription factor | 2,33 | 5,66E-15 | 2,64 | 3,07559E-10 |
| Nr4a3 | nuclear receptor subfamily 4, group A, member 3 | 2,33 | 1,98E-02 | 3,81 | 3,7224E-12 |
| Klf6 | Kruppel-like factor 6 | 2,5 | 4,85E-15 | 2,05 | 4,27739E-07 |
| Slc11a1 | solute carrier family 11 (proton-coupled divalent metal ion transporters), member 1 | 2,53 | 1,75E-10 | 2,82 | 0,001164602 |
| Stat3 | signal transducer and activator of transcription 3 | 2,56 | 9,34E-25 | 2,60 | 1,66724E-12 |
| Hmga2 | high mobility group AT-hook 2 | 2,60 | 1,31E-18 | 3,40 | 0,002863609 |
| Itga3 | integrin alpha 3 | 2,64 | 1,27E-03 | 2,06 | 0,001591151 |
| Il18 | interleukin 18 | 2,65 | 8,67E-06 | 2,43 | 0,009146839 |
| Mdfic | MyoD family inhibitor domain containing | 2,70 | 2,45E-12 | 2,25 | 6,39424E-14 |
| Brip1 | BRCA1 interacting protein C-terminal helicase 1 | 2,73 | 6,38E-13 | 2,21 | 2,17992E-06 |
| Tox2 | TOX high mobility group box family member 2 | 2,76 | 2,32E-12 | 2,38 | 0,001456075 |
| Tnfrsf1a | tumor necrosis factor receptor superfamily, member 1a | 2,82 | 2,18E-26 | 2,89 | 3,85969E-09 |
| Fstl3 | follistatin-like 3 | 2,84 | 4,75E-14 | 3,25 | 2,41436E-08 |
| Anxa7 | annexin A7 | 2,85 | 4,34E-28 | 2,09 | 6,10574E-08 |
| Nfkbid | nuclear factor of kappa light polypeptide gene enhancer in B cells inhibitor, delta | 2,95 | 6,30E-07 | 5,47 | 1,11452E-06 |
| Ahr | aryl-hydrocarbon receptor | 3,04 | 1,58E-30 | 2,93 | 4,66968E-16 |
| Nampt | nicotinamide phosphoribosyltransferase | 3,06 | 2,82E-30 | 2,64 | 5,28671E-12 |
| Atp8b1 | ATPase, class I, type 8B, member 1 | 3,17 | 1,53E-13 | 3,12 | 1,63129E-12 |
| Twist2 | twist basic helix-loop-helix transcription factor 2 | 3,18 | 2,07E-22 | 2,12 | 0,01154229 |
| Ptgfr | prostaglandin F receptor | 3,18 | 6,36E-03 | 2,53 | 0,000101028 |
| Runx1 | runt related transcription factor 1 | 3,18 | 2,72E-10 | 2,85 | 1,30824E-07 |
| Slit2 | slit homolog 2 (Drosophila) | 3,29 | 1,84E-20 | 4,40 | 5,43469E-06 |
| Tlr9 | toll-like receptor 9 | 3,37 | 7,79E-09 | 6,10 | 1,30055E-05 |
| Atad2 | ATPase family, AAA domain containing 2 | 3,44 | 3,36E-12 | 3,00 | 7,69494E-16 |
| Smad3 | SMAD family member 3 | 3,54 | 9,02E-20 | 2,62 | 7,33453E-06 |
| Cdk6 | cyclin-dependent kinase 6 | 3,56 | 9,57E-08 | 2,38 | 3,9933E-05 |
| Nfkb2 | nuclear factor of kappa light polypeptide gene enhancer in B cells 2, p49/p100 | 3,59 | 3,37E-35 | 4,17 | 1,58577E-25 |

| | | | | | |
|---|---|---|---|---|---|
| Tmem173 | transmembrane protein 173 | 3,61 | 6,46E-24 | 2,61 | 0,000558315 |
| Skil | SKI-like | 3,65 | 8,27E-31 | 2,38 | 5,71751E-06 |
| Tlr2 | toll-like receptor 2 | 3,73 | 2,33E-22 | 4,12 | 2,07157E-07 |
| Serpinb9 | serine (or cysteine) peptidase inhibitor, clade B, member 9 | 3,87 | 2,82E-09 | 3,22 | 1,91432E-05 |
| Tnfrsf10b | tumor necrosis factor receptor superfamily, member 10b | 3,88 | 8,44E-08 | 5,48 | 1,2247E-22 |
| Glis3 | GLIS family zinc finger 3 | 3,93 | 5,75E-07 | 2,48 | 0,00118371 |
| Trib1 | tribbles pseudokinase 1 | 3,93 | 1,33E-32 | 2,51 | 0,000180333 |
| Fhl2 | four and a half LIM domains 2 | 3,94 | 1,27E-32 | 2,27 | 4,75296E-07 |
| Relb | avian reticuloendotheliosis viral (v-rel) oncogene related B | 3,94 | 4,76E-38 | 3,71 | 6,03492E-33 |
| Cd44 | CD44 antigen | 4,01 | 1,43E-36 | 2,87 | 8,40137E-15 |
| Plk3 | polo-like kinase 3 | 4,11 | 3,11E-36 | 4,27 | 9,23557E-17 |
| Nupr1 | nuclear protein transcription regulator 1 | 4,13 | 1,23E-31 | 3,04 | 0,001415459 |
| Plscr1 | phospholipid scramblase 1 | 4,14 | 1,37E-31 | 4,02 | 2,75273E-13 |
| Fosb | FBJ osteosarcoma oncogene B | 4,17 | 1,03E-14 | 2,50 | 2,75806E-09 |
| Pawr | PRKC, apoptosis, WT1, regulator | 4,25 | 3,08E-17 | 2,57 | 0,000203418 |
| Wnt10a | wingless-type MMTV integration site family, member 10A | 4,25 | 3,61E-13 | 9,17 | 2,21255E-17 |
| Lrrc32 | leucine rich repeat containing 32 | 4,33 | 3,44E-70 | 3,73 | 4,95112E-11 |
| Vegfa | vascular endothelial growth factor A | 4,41 | 6,07E-45 | 2,28 | 8,77636E-05 |
| Pcgf5 | polycomb group ring finger 5 | 4,42 | 9,63E-33 | 3,83 | 4,51901E-26 |
| Smad7 | SMAD family member 7 | 4,43 | 1,82E-22 | 2,42 | 0,000106198 |
| Fos | FBJ osteosarcoma oncogene | 4,45 | 3,60E-38 | 2,68 | 1,45294E-07 |
| Tgif1 | TGFB-induced factor homeobox 1 | 4,51 | 3,87E-79 | 3,71 | 1,82438E-22 |
| Klf10 | Kruppel-like factor 10 | 4,58 | 1,23E-37 | 4,30 | 1,0843E-19 |
| Daxx | Fas death domain-associated protein | 4,63 | 5,28E-43 | 5,35 | 3,05745E-17 |
| Stc1 | stanniocalcin 1 | 4,64 | 6,33E-11 | 2,31 | 0,002520536 |
| Ccr5 | chemokine (C-C motif) receptor 5 | 4,70 | 1,48E-24 | 7,80 | 4,331E-07 |
| Atf5 | activating transcription factor 5 | 4,78 | 9,09E-54 | 4,86 | 3,74928E-13 |
| Nfkbia | nuclear factor of kappa light polypeptide gene enhancer in B cells inhibitor, alpha | 4,86 | 5,91E-39 | 3,01 | 1,47194E-05 |
| Nr1h3 | nuclear receptor subfamily 1, group H, member 3 | 4,98 | 2,33E-20 | 2,17 | 0,004106621 |
| Hes1 | hairy and enhancer of split 1 (Drosophila) | 5,13 | 1,76E-23 | 3,34 | 2,04765E-09 |
| Arid5a | AT rich interactive domain 5A (MRF1-like) | 5,21 | 9,68E-36 | 3,12 | 4,42175E-24 |
| Cebpg | CCAAT/enhancer binding protein (C/EBP), gamma | 5,21 | 4,02E-32 | 3,08 | 3,12405E-11 |
| F2rl1 | coagulation factor II (thrombin) receptor-like 1 | 5,25 | 4,56E-27 | 4,17 | 1,98446E-05 |
| Irf1 | interferon regulatory factor 1 | 5,27 | 1,97E-03 | 9,85 | 6,13646E-35 |
| Bhlhe40 | basic helix-loop-helix family, member e40 | 5,30 | 1,80E-77 | 2,88 | 1,93539E-05 |
| Hoxb9 | homeobox B9 | 5,42 | 9,23E-50 | 11,66 | 3,37922E-58 |
| Il33 | interleukin 33 | 5,66 | 1,02E-27 | 3,32 | 3,92711E-06 |
| Fosl2 | fos-like antigen 2 | 5,69 | 2,13E-82 | 5,75 | 4,47782E-25 |
| Pml | promyelocytic leukemia | 5,72 | 2,99E-25 | 5,31 | 1,04565E-45 |
| Arntl2 | aryl hydrocarbon receptor nuclear translocator-like 2 | 5,92 | 2,21E-15 | 3,84 | 5,03252E-07 |
| Csf1 | colony stimulating factor 1 (macrophage) | 6,09 | 2,42E-40 | 5,54 | 2,81091E-24 |
| Bcl6 | B cell leukemia/lymphoma 6 | 6,10 | 5,98E-30 | 2,31 | 4,93096E-05 |
| Cebpb | CCAAT/enhancer binding protein (C/EBP), beta | 6,17 | 4,48E-48 | 2,85 | 0,006042819 |

| | | | | | |
|---|---|---|---|---|---|
| Zfp36 | zinc finger protein 36 | 6,20 | 2,38E-95 | 4,29 | 2,39901E-18 |
| Cited2 | Cbp/p300-interacting transactivator, with Glu/Asp-rich carboxy-terminal domain, 2 | 6,31 | 5,26E-80 | 3,73 | 9,41158E-09 |
| Irf5 | interferon regulatory factor 5 | 6,55 | 3,13E-44 | 5,41 | 1,84498E-11 |
| Trim6 | tripartite motif-containing 6 | 6,73 | 5,80E-09 | 7,78 | 1,77033E-17 |
| Ncoa7 | nuclear receptor coactivator 7 | 6,75 | 4,93E-93 | 2,04 | 5,93745E-07 |
| Nr1h4 | nuclear receptor subfamily 1, group H, member 4 | 6,83 | 1,85E-05 | 3,38 | 0,000441343 |
| Noct | nocturnin | 6,98 | 3,31E-67 | 2,99 | 9,91041E-17 |
| Lef1 | lymphoid enhancer binding factor 1 | 6,98 | 2,93E-17 | 3,93 | 1,46723E-06 |
| Dusp5 | dual specificity phosphatase 5 | 7,02 | 3,92E-35 | 4,78 | 1,18189E-23 |
| Myc | myelocytomatosis oncogene | 7,13 | 5,82E-72 | 3,38 | 2,10193E-23 |
| Irf9 | interferon regulatory factor 9 | 7,24 | 1,10E-33 | 11,24 | 4,46792E-62 |
| Plaur | plasminogen activator, urokinase receptor | 7,32 | 2,26E-34 | 3,09 | 0,000188141 |
| Arhgef2 | rho/rac guanine nucleotide exchange factor (GEF) 2 | 7,40 | 1,33E-63 | 5,46 | 2,05032E-21 |
| Cebpd | CCAAT/enhancer binding protein (C/EBP), delta | 7,69 | 4,64E-116 | 4,69 | 4,9312E-08 |
| Atf3 | activating transcription factor 3 | 8,13 | 6,45E-65 | 4,99 | 5,47452E-19 |
| Batf3 | basic leucine zipper transcription factor, ATF-like 3 | 8,65 | 1,29E-34 | 6,90 | 1,79678E-17 |
| Stat1 | signal transducer and activator of transcription 1 | 9,20 | 5,82E-07 | 13,45 | 9,0428E-126 |
| Nfkbie | nuclear factor of kappa light polypeptide gene enhancer in B cells inhibitor, epsilon | 9,43 | 6,30E-61 | 7,44 | 7,75899E-41 |
| Bcl3 | B cell leukemia/lymphoma 3 | 9,43 | 3,40E-65 | 5,53 | 1,36978E-27 |
| Lgals9 | lectin, galactose binding, soluble 9 | 10,39 | 3,67E-46 | 9,44 | 1,803E-24 |
| Runx2 | runt related transcription factor 2 | 10,90 | 8,17E-20 | 6,67 | 8,92795E-13 |
| Tnc | tenascin C | 11,00 | 8,27E-111 | 13,44 | 5,77657E-58 |
| Maff | v-maf musculoaponeurotic fibrosarcoma oncogene family, protein F (avian) | 11,80 | 2,18E-115 | 7,17 | 2,94135E-20 |
| Egr1 | early growth response 1 | 12,57 | 4,46E-193 | 9,98 | 1,26304E-61 |
| Ddit3 | DNA-damage inducible transcript 3 | 12,65 | 9,42E-76 | 5,56 | 1,49929E-10 |
| Nfil3 | nuclear factor, interleukin 3, regulated | 13,56 | 7,69E-101 | 5,49 | 2,48454E-15 |
| Helz2 | helicase with zinc finger 2, transcriptional coactivator | 14,24 | 4,39E-52 | 21,07 | 3,13212E-84 |
| Lck | lymphocyte protein tyrosine kinase | 14,91 | 1,08E-36 | 6,91 | 0,000104289 |
| Mndal | myeloid nuclear differentiation antigen like | 16,18 | 1,33E-17 | 18,10 | 1,74483E-24 |
| Tlr3 | toll-like receptor 3 | 16,58 | 1,07E-115 | 12,23 | 2,81941E-40 |
| Serpine1 | serine (or cysteine) peptidase inhibitor, clade E, member 1 | 17,11 | 2,33E-13 | 6,21 | 3,38861E-11 |
| Tfap2a | transcription factor AP-2, alpha | 18,35 | 4,02E-23 | 13,73 | 1,47763E-23 |
| Batf2 | basic leucine zipper transcription factor, ATF-like 2 | 19,72 | 1,51E-06 | 28,28 | 1,05768E-39 |
| Ddx58 | DEAD (Asp-Glu-Ala-Asp) box polypeptide 58 | 19,82 | 5,82E-27 | 19,85 | 5,9873E-147 |
| Ucn2 | urocortin 2 | 19,98 | 3,23E-51 | 45,07 | 1,84265E-07 |
| Slfn1 | schlafen 1 | 20,53 | 8,17E-05 | 152,54 | 2,40797E-14 |
| Ifi203 | interferon activated gene 203 | 20,62 | 6,97E-20 | 24,76 | 1,72926E-32 |
| Calca | calcitonin/calcitonin-related polypeptide, alpha | 22,78 | 4,81E-05 | 12,75 | 2,1416E-06 |
| Lif | leukemia inhibitory factor | 24,23 | 5,81E-158 | 23,94 | 7,2581E-101 |
| Tnfsf8 | tumor necrosis factor (ligand) superfamily, member 8 | 33,90 | 3,03E-03 | 5,41 | 2,54052E-25 |
| Rorb | RAR-related orphan receptor beta | 36,16 | 2,91E-10 | 15,49 | 4,45562E-16 |
| Tfap2b | transcription factor AP-2 beta | 36,84 | 1,80E-15 | 22,75 | 1,74538E-13 |
| Ifi204 | interferon activated gene 204 | 37,86 | 3,67E-32 | 43,50 | 7,63861E-42 |

| | | | | | |
|---|---|---|---|---|---|
| Stc2 | stanniocalcin 2 | 41,36 | 1,14E-89 | 5,07 | 3,7877E-05 |
| Irf7 | interferon regulatory factor 7 | 93,03 | 3,04E-13 | 251,37 | 1,1427E-142 |
| Shh | sonic hedgehog | 102,82 | 6,77E-05 | 267,80 | 3,80132E-14 |
| Gdnf | glial cell line derived neurotrophic factor | 104,43 | 3,28E-261 | 47,26 | 1,83713E-27 |
| Fosl1 | fos-like antigen 1 | 114,02 | 2,12E-123 | 5,53 | 7,99526E-06 |
| Ptprn | protein tyrosine phosphatase, receptor type, N | 114,74 | 1,36E-173 | 24,08 | 1,99716E-44 |
| Nrg1 | neuregulin 1 | 128,63 | 4,57E-12 | 20,73 | 5,29674E-36 |
| Trib3 | tribbles pseudokinase 3 | 131,41 | 4,15E-52 | 62,52 | 4,16474E-51 |
| Trim66 | tripartite motif-containing 66 | 155,01 | 4,44E-18 | 39,18 | 5,73698E-56 |
| Ccl5 | chemokine (C-C motif) ligand 5 | 1253,15 | 6,74E-87 | 188,95 | 2,67834E-29 |

**Table S2**: List of 180 transcriptional regulators found in common in RNA-seq 1 and in RNA-seq 2 data sets (with FC >2); gene symbol, gene name along with fold-change and P-value are indicated for each data set. Of note, only three transcripts were found to be upregulated in one set and downregulated in the other. The remaining discordant transcripts were found to have a FC ≤ 2 in one data set and to be only modestly deregulated (with a FC ≤ 3) in the other.

**Table S3:** List of 52 transcriptional regulators with Fold-change FC >5.

| | | 1st RNA-seq | | 2nd RNA-seq | | Pubmed search with key word | | Encoded protein/ expected non-cell or cell autonomous function | Interferome | Repair | Mda5/Mavs pathway |
|---|---|---|---|---|---|---|---|---|---|---|---|
| **Gene symbol** | **Full name** | **Fold change** | **P-value** | **Fold change** | **P-value** | **Neural crest** | **Schwann cells (SCs)** | | **yess/no** | **yes/no** | |
| Chp2 | calcineurin-like EF hand protein 2 | 0,13 | 2,03E-34 | 0,07 | 6,84718E-37 | nr | nr | small calcium-binding protein | no | yes | |
| Abca2 | ATP-binding cassette, sub-family A (ABC1), member 2 | 0,14 | 2,2E-39 | 0,22 | 1,37891E-09 | nr | yes, PMID 17240058 + PMID 36385764 showing expression in CNS neurons and association with neurodegeneration | membrane-associated protein, cell automomous | yes | yes | |
| Lmo1 | LIM domain only 1 | 0,17 | 0,0000928 | 0,19 | 2,63034E-09 | neuroblastoma, PMID 33779609 + PMID 37183825 showing adrenergic cell identity of neuroblastoma requires LMO1 | nr | transcriptional cofactor, non-cell autonomous | yes | yes | |
| Cdh1 | cadherin 1 | 0,20 | 0,0132 | 0,18 | 8,95745E-06 | yes, including PMID 37225711, PMID 27126000 + PMID 37183825 (craniofacial) | affect myelin in CNS, PMID 14716021 | cell adhesion protein | yes | yes | |
| Rxrg | retinoid X receptor gamma | 0,23 | 8,93E-23 | 0,18 | 1,1578E-15 | migrating cranial neural crest cells + differentiating neurogenic derivatives (drg) | expressed in MPNST PMID: 29131833; downregulated upon nerve repair PMID: 29867349 ; RXRg-SREBP-Lipid Biosynthesis Axis control myelination in Schwann Cells PMID: 25310982 | Nuclear Receptor, DNA binding receptor, negativey regulate Tfap2a expression PMID: 33774011 | yes | yes | |
| Irf1 | interferon regulatory factor 1 | 5,27 | 0,00198 | 9,85 | 6,13646E-35 | nr | oligodendrocyte injury and inflammatory demyelination in MS and EAE, PMID 22499673 | transcriptional regulator, no direct link with Egr1 or Tfap2a | yes | no | yes |
| Hoxb9 | homeobox B9 | 5,42 | 9,23E-50 | 11,66 | 3,37922E-58 | nr | nr | transcription factor | no | no | |
| Fosl2 | fos-like antigen 2 | 5,69 | 2,13E-82 | 5,75 | 4,47782E-25 | nr | activated after 7 days in repair PMID: 34744629 | part of transcription factor complex AP-1 | yes | yes | |
| Pml | promyelocytic leukemia | 5,72 | 2,99E-25 | 5,31 | 1,04565E-45 | sympathetic nervous system, PMID 28148693 | Facial nerve schwannomas, PMID 32913501 | phosphoprotein localizes to nuclear bodies where it functions as a transcription factor | yes | no | |
| Csf1 | colony stimulating factor 1 (macrophage) | 6,09 | 2,42E-40 | 5,54 | 2,81091E-24 | expressed in iridophores and enteric neuron, PMID 27521479 | SCs bearing phagocytic phenotypes, PMID 31859421 + PMID 23737760 showing demyelination-induced reactive astrocytes express numerous ligands including Csf1 that act on homeostatic as well as activated microglia | cytokine, cell autonomous | yes | yes | |
| Irf5 | interferon regulatory factor 5 | 6,55 | 3,13E-44 | 5,41 | 1,84498E-11 | nr | nr | transcription factor | yes | yes | yes |
| Trim6 | tripartite motif-containing 6 | 6,73 | 5,8E-09 | 7,78 | 1,77033E-17 | nr | nr | E3 ubiquitin ligase | yes | no | |
| Irf9 | interferon regulatory factor 9 | 7,24 | 1,1E-33 | 11,24 | 4,46792E-62 | nr | nr | transcription factor | yes | no | yes |
| Arhgef2 | Rho guanine nucleotide exchange factor (GEF) 28 | 7,4 | 1,33E-63 | 5,46 | 2,05032E-21 | lfc increases ectodermal competence for neural induction and reduced NC formation PMID: 10525189 | nr | Rho GTPase; cytoplasmic | yes | no | |
| Batf3 | basic leucine zipper transcription factor, ATF-like 3 | 8,65 | 1,29E-34 | 6,90 | 1,79678E-17 | nr | nr | transcription factor, JUN interactor | yes | no | |

| | | | | | | | | | | | |
|---|---|---|---|---|---|---|---|---|---|---|---|
| Stat1 | signal transducer and activator of transcription 1 | 9,20 | 0,000000582 | 13,45 | 9,0428E-126 | nr | yes, regulate oct6 expression, PMID 20687925 + The transcription factor Stat-1 is essential for Schwann cell differentiation, myelination and myelin sheath regeneration PMID 37365519 | Signal transducer and transcription activator, cell autonomous | yes | no | yes |
| Bcl3 | B cell leukemia/lymphoma 3 | 9,43 | 3,4E-65 | 5,53 | 1,36978E-27 | nr | nr | transcriptional co-activator | no | yes | |
| Nfkbie | nuclear factor of kappa light polypeptide gene enhancer in B cells inhibitor, epsilon | 9,43 | 6,3E-61 | 7,44 | 7,75899E-41 | nr | nr | Inhibits NF-kappa-B by complexing with and trapping it in the cytoplasm | yes | no | |
| Lgals9 | lectin, galactose binding, soluble 9 | 10,39 | 3,67E-46 | 9,44 | 1,803E-24 | nr | nr | cell-cell and cell-matrix interactions | yes | yes | |
| Runx2 | runt related transcription factor 2 | 10,90 | 8,17E-20 | 6,67 | 8,92795E-13 | Osteoblastic differentiation and skeletal morphogenesis, including PMID 32417535, PMID 38063851 | Schwann cell differentiation and migration after sciatic nerve injury PMID 30374602, PMID 34744629, PMID, 38051902 + regulated by c-Jun- PMID 25614629, but not in SC PMID: 27581455 + PMID 34902518 showing that curcumin promoted Schwann cell proliferation and myelination through Runx2 | transcription factor, cell automomous + RUNX2 and EGR1 co-repressed Htra1 and increased the expression levels of other osteoblast marker genes, such as osterix, osteocalcin PMID 32324256 | yes | yes | |
| Tnc | tenascin C | 11,00 | 8,27E-111 | 13,44 | 5,77657E-58 | yes, PMID 23958436 and references therein | PMID: 38951719 expression of Tnc in sciatic nerve; PMID 26497118 nerve fibroblasts accumulating at the lesion site + PMID 35681468 Tnc has an inhibitory effect on the differentiation of OPCs and the remyelination efficiency of oligodendrocytes. | extracellular matrix glycoprotein, non cell autonomous | yes | yes | |
| Maff | v-maf musculoaponeurotic fibrosarcoma oncogene family, protein F (avian) | 11,80 | 2,18E-115 | 7,17 | 2,94135E-20 | nr | nr | transcription factor | yes | no | |
| Egr1 | early growth response 1 | 12,57 | 4,46E-193 | 9,98 | 1,26304E-61 | cranial cartilage PMID 23209659 | yes, during development PMID 9418958 and upon injury PMID 27058953, myelin inhibitor | transcription factor, cell autonomous | yes | yes | |
| Ddit3 | DNA-damage inducible transcript 3 | 12,65 | 9,42E-76 | 5,56 | 1,49929E-10 | nr | Ablation of Chop, from POS63del mice rescues their motor deficit and reduces active demyelination PMID 23547100 | transcription factor, cell autonomous, regulate Egr1 expression PMID 22496745 | yes | no | PERK signalling |
| Nfil3 | nuclear factor, interleukin 3, regulated | 13,56 | 7,69E-101 | 5,49 | 2,48454E-15 | nr | mutation lead to sensory autonomic neuropathy | transcriptional regulator, non cell autonomous | yes | yes | |
| Helz2 | helicase with zinc finger 2, transcriptional coactivator | 14,24 | 4,39E-52 | 21,07 | 3,13212E-84 | nr | nr | transcriptional co-activator | yes | no | yes |
| Lck | lymphocyte protein tyrosine kinase | 14,91 | 1,08E-36 | 6,91 | 0,000104289 | nr | Lck mediates β1-integrin signalling to regulate Schwann cell migration and myelination , PMID 23715271 | Src family of protein tyrosine kinase, cell autonomous | yes | no | - |

| | | | | | | | | | | | |
|---|---|---|---|---|---|---|---|---|---|---|---|
| Mndal | myeloid nuclear differentiation antigen like | 16,18 | 1,33E-17 | 18,10 | 1,74483E-24 | nr | nr | ISG | yes | no | |
| Tlr3 | toll-like receptor 3 | 16,58 | 1,07E-115 | 12,23 | 2,81941E-40 | nr | yes, all tlr PMID 29113341 | single transmembrane cell-surface receptor, cell autonomous | yes | no | |
| Serpine1 | serine (or cysteine) peptidase inhibitor, clade E, member 1 | 17,11 | 2,33E-13 | 6,21 | 3,38861E-11 | nr | nr | serine proteinase inhibitor | yes | no | |
| Tfap2a | transcription factor AP-2, alpha | 18,35 | 4,02E-23 | 13,73 | 1,47763E-23 | yes, PMID 29852131 Tfap2a in early NCC + PMID 37398373 PMID 38063857 Tfap paralogs control craniofacial development through Alx + PMID 36563566 Zebrafish anterior segment mesenchyme progenitors are defined by function of tfap2a | yes, PMID 11553286, PMID 31650361, PMID 39009095 myelin inhibitor | transcription factor, cell autonomous | yes | yes | |
| Batf2 | basic leucine zipper transcription factor, ATF-like 2 | 19,72 | 0,00000151 | 28,28 | 1,05768E-39 | nr | nr | AP1 transcription factor familly | yes | no | |
| Ddx58 | DEAD (Asp-Glu-Ala-Asp) box polypeptide 58 | 19,82 | 5,82E-27 | 19,85 | 5,9873E-147 | nr | nr | viral double-stranded (ds) RNA recognition RNA Helicase RIG-I | yes | yes | |
| Ucn2 | urocortin 2 | 19,98 | 3,23E-51 | 45,07 | 1,84265E-07 | nr | nr | endogenous ligand for CRF type 2 receptor | no | yes | |
| Slfn1 | schlafen 1 | 20,53 | 0,0000817 | 152,54 | 2,40797E-14 | nr | nr | ISG | yes | yes | |
| Ifi203 | interferon activated gene 203 | 20,62 | 6,97E-20 | 24,76 | 1,72926E-32 | nr | nr | ISG | yes | no | |
| Calca | calcitonin/calcitonin-related polypeptide, alpha | 22,78 | 0,0000481 | 12,75 | 2,1416E-06 | affected in frontal bone of dlx3 mutant, PMID 22886599 + PMID 37044067 sensory neuron marker | nr | peptide hormones calcitonin | yes | no | |
| Lif | leukemia inhibitory factor | 24,23 | 5,81E-158 | 23,94 | 7,2581E-101 | NSC reprogrammation | PMID: 10386959, expressed in Schwann cells after rat sciatic nerve injury. Affect proliferation and migration ability of Schwann cells | cytokine, cell autonomous | yes | yes | |
| Tnfsf8 | tumor necrosis factor (ligand) superfamily, member 8 | 33,90 | 0,00304 | 5,41 | 2,54052E-25 | nr | nr | cytokine | yes | no | |
| Rorb | RAR-related orphan receptor beta | 36,16 | 2,91E-10 | 15,49 | 4,45562E-16 | nr | nr | nuclear hormone receptors | no | yes | |
| Tfap2b | transcription factor AP-2 beta | 36,84 | 1,8E-15 | 22,75 | 1,74538E-13 | PMID 31848212, pair with tfap2a during NC differentiation | nr | transcription factor | no | no | |
| Ifi204 | interferon activated gene 204 | 37,86 | 3,67E-32 | 43,50 | 7,63861E-42 | nr | nr | ISG | yes | no | |
| Stc2 | stanniocalcin 2 | 41,36 | 1,14E-89 | 5,07 | 3,7877E-05 | nr | nr | secreted, homodimeric glycoprotein | no | yes | |
| Irf7 | interferon regulatory factor 7 | 93,03 | 3,04E-13 | 251,37 | 1,1427E-142 | nr | myelin alterations are associated to IRF7 deregulation during acute demyelination, PMID 19121307 | TF, cell autonomous | yes | yes | yes |
| Shh | sonic hedgehog | 102,82 | 0,0000677 | 267,80 | 3,80132E-14 | various NCC derivatives | PMID 27581455 activated during SC repair, but not necessary for myelin formation PMID: 38771121 | secreted protein, cell autonomous | no | yes | |

| | | | | | | | | | | | |
|---|---|---|---|---|---|---|---|---|---|---|---|
| Gdnf | glial cell line derived neurotrophic factor | 104,43 | 3,28E-261 | 47,26 | 1,83713E-27 | various NCC derivatives | PMID 32562261 Gdnf induces migration of Schwann cell precurosors. Role in repair PMID: 38995011 and pain sensitivity PMID: 38258905 | secreted ligand of the TGF-beta familly, cell autonomous | no | yes | |
| Fosl1 | fos-like antigen 1 | 114,02 | 2,12E-123 | 5,53 | 7,99526E-06 | FOSL1 causes melanocyte reprogramming and transformation and induces neuronal genes PMID: 28481878 | activated early in repair PMID: 34744629, but not in SC PMID: 27581455. PMID 37949219 modulates Schwann cell responses in the wound microenvironment and regulates peripheral nerve regeneration | AP1 trascription factor subnit, leucine zipper proteins that can dimerize with proteins of the JUN family | yes | yes | |
| Ptprn | protein tyrosine phosphatase, receptor type, N | 114,74 | 1,36E-173 | 24,08 | 1,99716E-44 | nr | nr | protein tyrosine phosphatase | no | no | |
| Nrg1 | neuregulin 1 | 128,63 | 4,57E-12 | 20,73 | 5,29674E-36 | PMID 17432114, several neurocristopathies | PMID: 31229498, role in SC development and repair | ligand for ERBB3 and ERBB4, non cell autonomous | yes | no | |
| Trib3 | tribbles pseudokinase 3 | 131,41 | 4,15E-52 | 62,52 | 4,16474E-51 | nr | PMID 27977799 in CNS repair | protein kinase | yes | no | |
| Trim66 | tripartite motif-containing 66 | 155,01 | 4,44E-18 | 39,18 | 5,73698E-56 | nr | nr | transcriptional repressor | no | no | |
| Ccl5 | chemokine (C-C motif) ligand 5 | 1253,15 | 6,74E-87 | 188,95 | 2,67834E-29 | nr | PMID 9521605 and PMID 36611893 showing gene is expressed in E12.5 DRG, Schwann cells, spinal cord and skin following infection | chemoattractant | yes | yes | |

**Table S3:** List of 52 transcriptional regulators with FC >5; gene symbol, full name, Fold change (FC) and P-value in each RNA-seq are indicated. Pubmed search colums indicate references (PMID) found upon use of "Neural crest" or "Schwann cells" key words. nr: not reported; Encoded protein/ expected non-cell or cell autonomous function column indicates main functions/localisation and PMID references (Columns indicated in green show genes encoding membrane-associated proteins, cytokine, or neurotrophic factors, and /or genes with a main function in sensory neurons). Interferome column indicates if genes are within the interferome database; Repair column shows RNAs in common with (Kim et al.,2012-Arthur-Farraj et al.,2017). Network analysis and interferome confirm central role of IFN signaling,. Last column (and blue color) indicates the genes previously described within Mda5/Mavs pathway.

1

**Table S4:** List of 174 genes identified in common among EGR1and EGR2 ChIPseq datasets and current *Adar1*cKO RNA-seq

| | EGR2 mutant | *Adar1*cKO mutant |
|---|---|---|
| CHDH | | |
| MBP | | |
| PLLP | | |
| KCNK1 | | |
| MME | | |
| ELOVL7 | | |
| FA2H | | |
| SLC6A1 | | |
| DUSP15 | | |
| GJB1 | | |
| ELOVL6 | | |
| HMGCS1 | | |
| FRZB | | |
| IDI1 | | |
| GPM6A | | |
| FDPS | | |
| LSS | | |
| SECISBP2L | | |
| EFHD1 | | |
| CHST2 | | |
| MT3 | | |
| ALDOC | | |
| MAL | | |
| DHCR24 | | |
| ACSS2 | | |
| TM7SF2 | | |
| PCYT2 | | |
| APOBEC2 | | |
| SLC7A2 | | |
| NSDHL | | |
| PRX | | |
| ACAT2 | | |
| SCN3B | | |
| HMGCR | | |
| GPR155 | | |
| SEMA5A | | |
| IL16 | | |
| TNNI2 | | |
| CASQ1 | | |
| ENO3 | | |
| PGAM2 | | |
| CNTN1 | | |
| SC5D | | |
| FDFT1 | | |
| CKM | | |
| MBOAT1 | | |
| SNCG | | |
| NEK1 | | |
| TMEM38A | | |
| ABCA2 | | |
| TESK2 | | |
| DHCR7 | | |
| NDRG1 | | |
| CD9 | | |
| SH3BGR | | |
| TPM2 | | |
| RAB40B | | |
| TMEM40 | | |
| HSD17B7 | | |
| TTN | | |
| RYR1 | | |
| AACS | | |
| MAPK8IP1 | | |
| ACSL1 | | |
| S100B | | |

| | | |
|---|---|---|
| ELOVL1 | | |
| PMVK | | |
| BMP7 | | |
| SQLE | | |
| ST6GAL1 | | |
| TBXA2R | | |
| SEMA3B | | |
| SLC6A15 | | |
| MRAS | | |
| LDLR | | |
| EXTL1 | | |
| PANK3 | | |
| CASKIN2 | | |
| AK3 | | |
| GLOD4 | | |
| ADAM10 | | |
| VWF | | |
| SIRT2 | | |
| PACS2 | | |
| UTRN | | |
| CD55 | | |
| TM6SF1 | | |
| MGST3 | | |
| RNF13 | | |
| STARD4 | | |
| LIMK2 | | |
| PIP4K2A | | |
| SLC20A2 | | |
| LIMCH1 | | |
| EPDR1 | | |
| EPHB6 | | |
| DNAJA4 | | |
| RAP1GDS1 | | |
| TRIM59 | | |
| CCNA2 | | |
| NTM | | |
| GPC1 | | |
| NR4A1 | | |
| MKI67 | | |
| PLK4 | | |
| DCK | | |
| PLEKHO1 | | |
| RAB32 | | |
| INCENP | | |
| MCM6 | | |
| NCAPD2 | | |
| AURKB | | |
| DNMT1 | | |
| MCM2 | | |
| DCLK1 | | |
| RACGAP1 | | |
| CEP55 | | |
| MIDN | | |
| TOP2A | | |
| ECT2 | | |
| RBL1 | | |
| BUB1 | | |
| ZWILCH | | |
| RAD51AP1 | | |
| MCM3 | | |
| SORCS2 | | |
| BRCA1 | | |
| RRM2 | | |
| FOS | | |
| MCM5 | | |
| LRRFIP1 | | |
| CD44 | | |
| KIF2C | | |
| SPATA13 | | |
| PLK2 | | |
| CDCA5 | | |
| ALCAM | | |

| | | |
|---|---|---|
| HMGA2 | | |
| UHRF1 | | |
| COL18A1 | | |
| KLF10 | | |
| ATF3 | | |
| THBS2 | | |
| PHLDA1 | | |
| RUNX2 | | |
| TNFRSF12A | | |
| LGALS3 | | |
| TRIM25 | | |
| PARP14 | | |
| HMGA1 | | |
| GPR37 | | |
| ABHD3 | | |
| GRIA2 | | |
| SLAIN1 | | |
| SPOCK1 | | |
| CUEDC2 | | |
| CSRP2 | | |
| WASF1 | | |
| SH3GL3 | | |
| CDKN1C | | |
| RNF103 | | |
| TMPRSS5 | | |
| POU3F1 | | |
| BZW2 | | |
| GSTA4 | | |
| LPCAT1 | | |
| ICK | | |
| PPP1R14C | | |
| PDGFC | | |
| CADM1 | | |
| TMEM189 | | |
| ME1 | | |
| SLIT2 | | |
| SPP1 | | |

**Table S4**: List of 174 transcriptional regulators evidenced in Chip-seq of EGR1, EGR2 and deregulated in current *Adar1*cKO RNA-seq;. Red : up-regulated and green : down-regulated in hypomorphic Egr2 mutant or upon Adar1 invalidation in NC compared to controls.

## Supplemental Figures

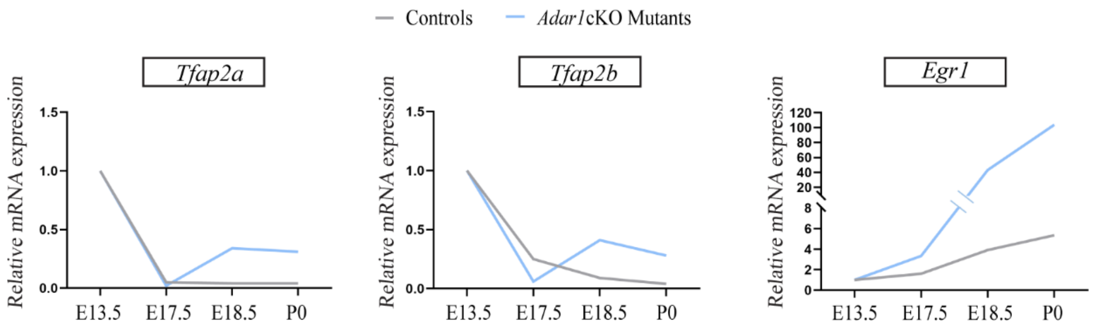


**Figure S1. Expression level of *Tfap2a, Tfap2b* and *Egr1* in DRG or sciatic nerves isolated from controls and *Adar1*cKO mutants at E13.5, E17.5, E18.5 and birth (new born, P0).** The relative abundance values of each transcripts was normalized to the internal control β-actin, and expression level in controls and *Adar1*cKO mutants represented relative to level quantified at E13.5. Note that although expressed at similar levels in E13.5 DRG of wild-type and *Adar1*cKO mutants, *Tfap2a* and *Tfap2b* are drastically downregulated from E17.5 onwards in controls (grey), but aberrantly re-expressed from E18.5 onwards in *Adar1*cKO mutants (blue). In contrast, *Egr1* expression is maintained at all stages analyzed in controls, but strikingly upregulated from E18.5 onwards in *Adar1*cKO mutants.

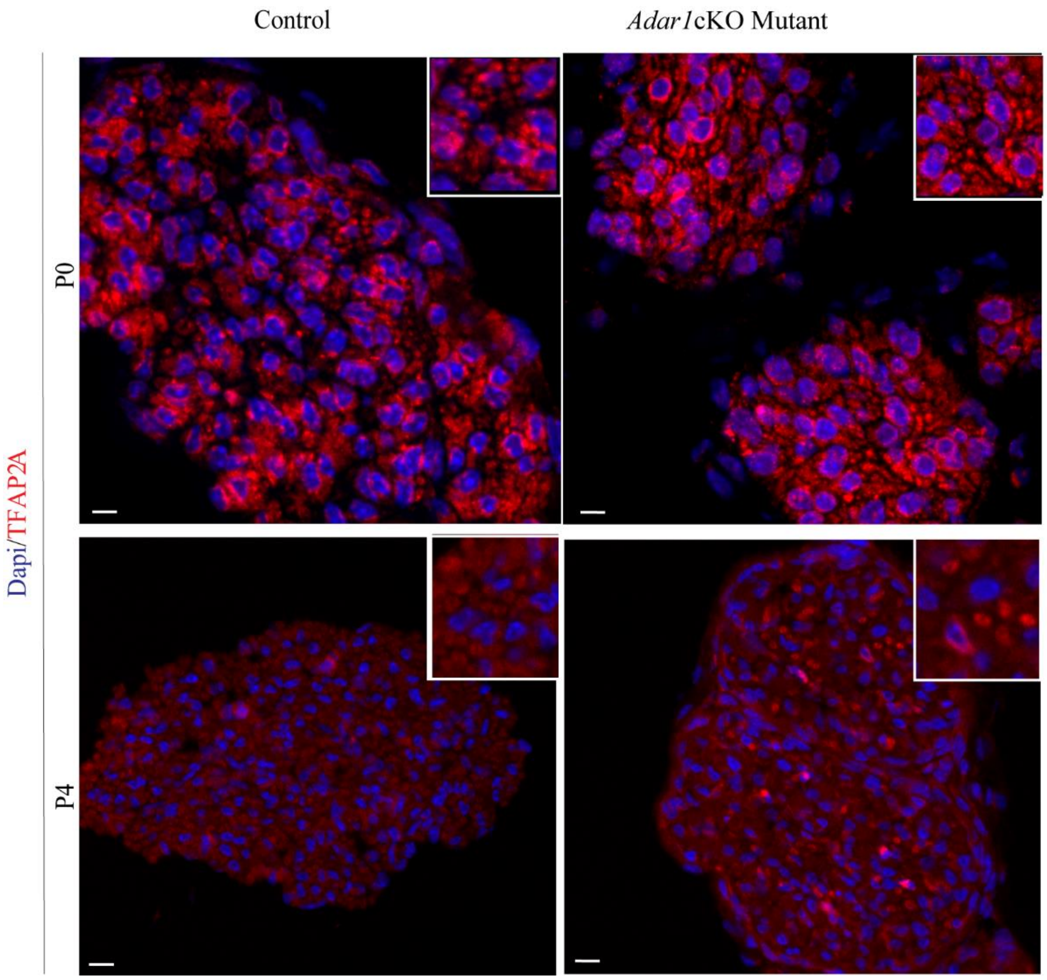


**Figure S2. Expression of TFAP2A protein in the sciatic nerves of *Adar1*cKO compared to controls at P0 and P4.** TFAP2A (red) immunostainings were performed on sections of sciatic nerves of *Adar1*cKO and controls mice at P0 and P4. Counter staining with DAPI is shown to identify nuclei. Scale bar: 60 µm at P0 and 30 µm at P4 .

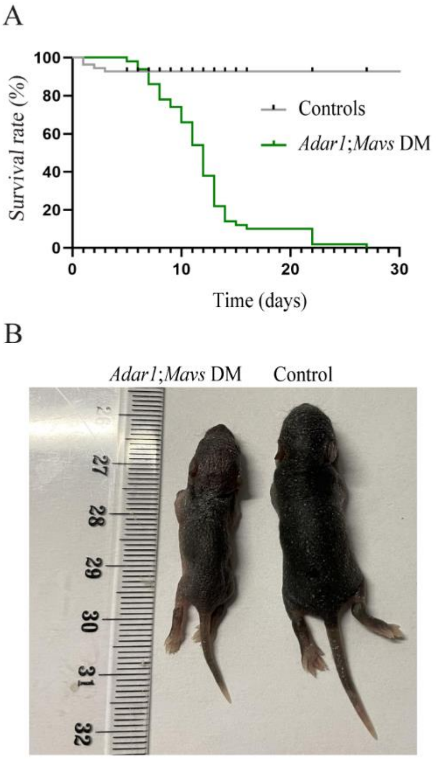


**Figure S3. Survival curve and pigmentation of *Adar1;Mavs* DM versus controls. (A)** Survival rate (%) of controls (grey), and *Adar1;Mavs* DM (green) over time (days) and **(B)** picture of *Adar1;Mavs* DM (left) and controls (right) showing normal pigmentation of animals of both genotypes.

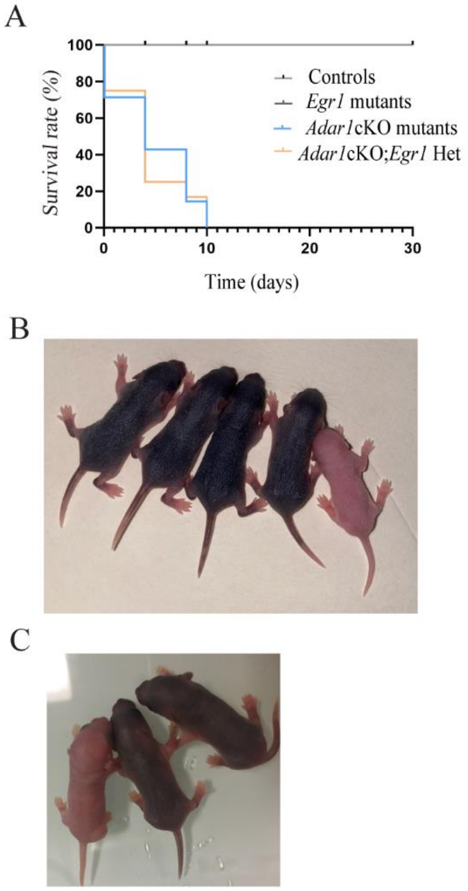


**Figure S4. Survival curve and pigmentation of *Adar1*cKO;*Egr1*Het and *Adar1*cKO mutants versus controls (A)** Survival rate (%) of controls (light grey), Egr1 homozygous (grey), *Adar1*cKO (blue) and *Adar1*cKO;*Egr1*Het (orange) over time (days). Note that deletion of one copy of *Egr1* does not rescue survival rate of *Adar1*CKO animals. **(B)** Picture of *Adar1*cKO;*Egr1*Het (right) compared to controls and (C) Picture of *Adar1*cKO (left) compared to controls. Note total depigmentation of *Adar1*cKO;*Egr1*Het and *Adar1*cKO mutants, demonstrating absence of pigmentation rescue upon deletion of one allele of *Erg1*.
